# Histologically Verified Dupuytren′s Disease in a 6‐Year‐Old: A Case Report and Literature Review

**DOI:** 10.1155/cro/9692425

**Published:** 2026-06-27

**Authors:** David Máška, Martin Vlach

**Affiliations:** ^1^ Department of Orthopedics, Second Faculty of Medicine, Charles University and Motol and Homolka University Hospital, Prague, Czech Republic

**Keywords:** case report, Dupuytren′s contracture, palmar fibromatosis, pediatrics

## Abstract

Dupuytren′s disease typically affects adults and only rarely occurs in children. We present a case of a 6‐year‐old boy with histologically confirmed palmar fibromatosis. Although uncommon, Dupuytren′s disease should be included in the differential diagnosis for palmar lesions in the pediatric population.

## 1. Introduction

Dupuytren′s disease, also known as palmar fibromatosis or Dupuytren′s contracture, is a common hereditary benign proliferative disease. Risk factors for its development include Caucasian race, male sex, Northern European descent, liver disease, alcoholism, smoking, diabetes mellitus, epilepsy, and previous hand trauma. Typically, it presents as a nodule or a cord on the palmar aspect of the hand, often contracting fingers into varying degrees of flexion. Despite affecting predominantly adults, there are sporadic reports of this disease in the pediatric population.

## 2. Case Report

An otherwise healthy boy of 6 years of age presented with a nodule in his right palm that had been present for 9 months. According to his mother, the appearance of the nodule seemed to correspond with a previous hand trauma, which occurred when the patient hit his friend with his right fist. The boy often played video games with a vibrating gamepad. There was no known family history of Dupuytren′s disease. The nodule became painful 7 months after appearing, that is, 2 months before his presentation to our clinic. There was no relevant family history of tumors, nodules, hand contractures, Dupuytren′s disease, or other forms of fibromatoses on either side of the patient’s family according to his mother.

On examination there was a mass of 10 × 10 × 5 mm in the middle of the palm that was tender to touch; there was no decrease in range of motion of the fingers or the wrist, no contractures, and no palpable cords. Ultrasound examination revealed a nonvascularized 7 × 10 × 4 mm encapsulated hypoechoic mass associated with a palmar flexor tendon, which was interpreted as being most consistent with a ganglion cyst.

Given the tenderness of the lesion, limiting the daily use of the hand, surgery was recommended. The incision was made directly above the mass in line with the palmar crease; subcutaneous layers were dissected until a nodule arising from the palmar aponeurosis was identified. A V‐shaped cutaneous flap was elevated for better exposure, and the nodule, which appeared different from a flexor tendon sheath ganglion cyst, was removed in toto, using a limited palmar fasciectomy. The patient was discharged the following day.

**Figure 1:**
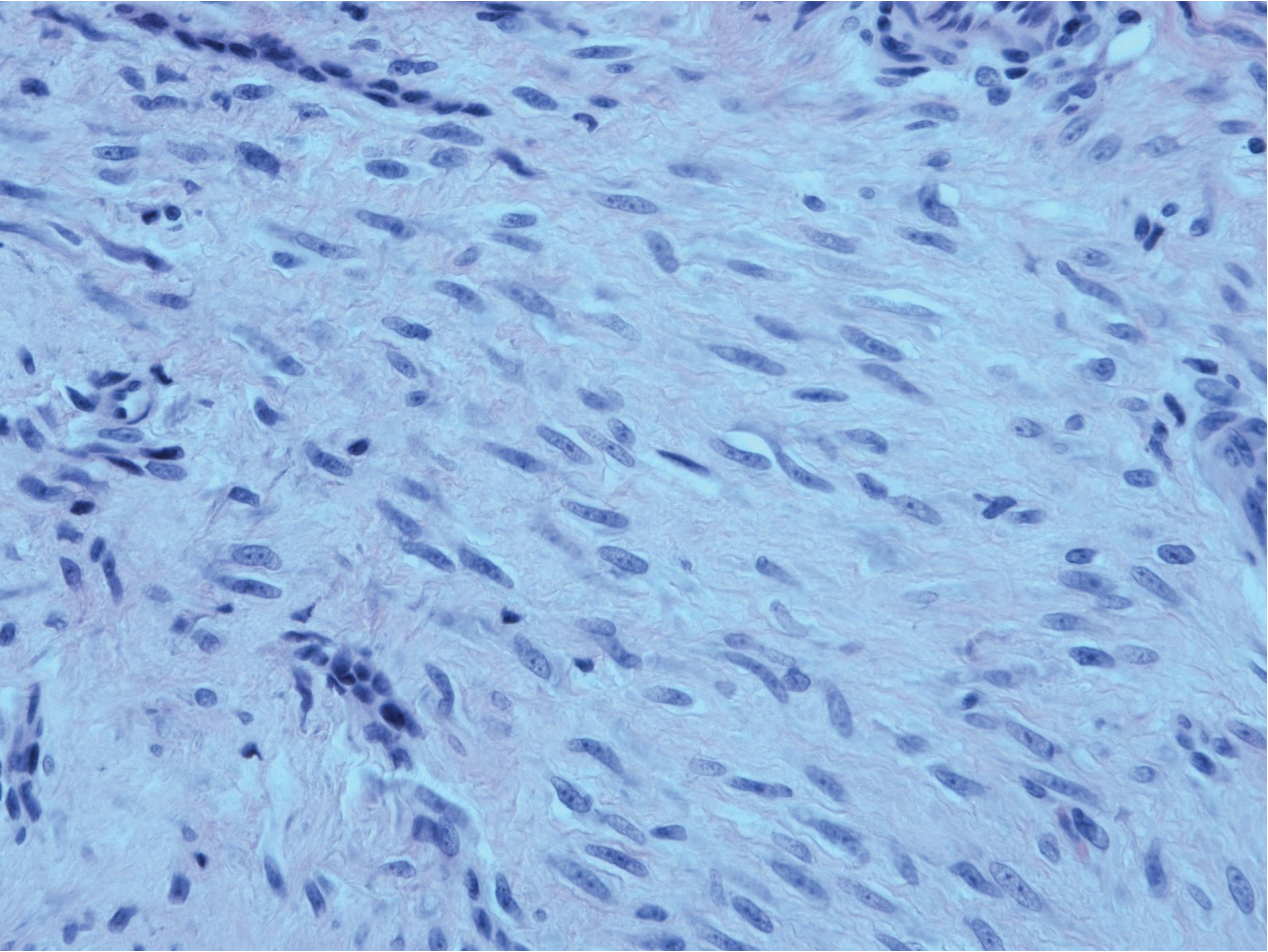
Hematoxylin and eosin staining—detail.

**Figure 2:**
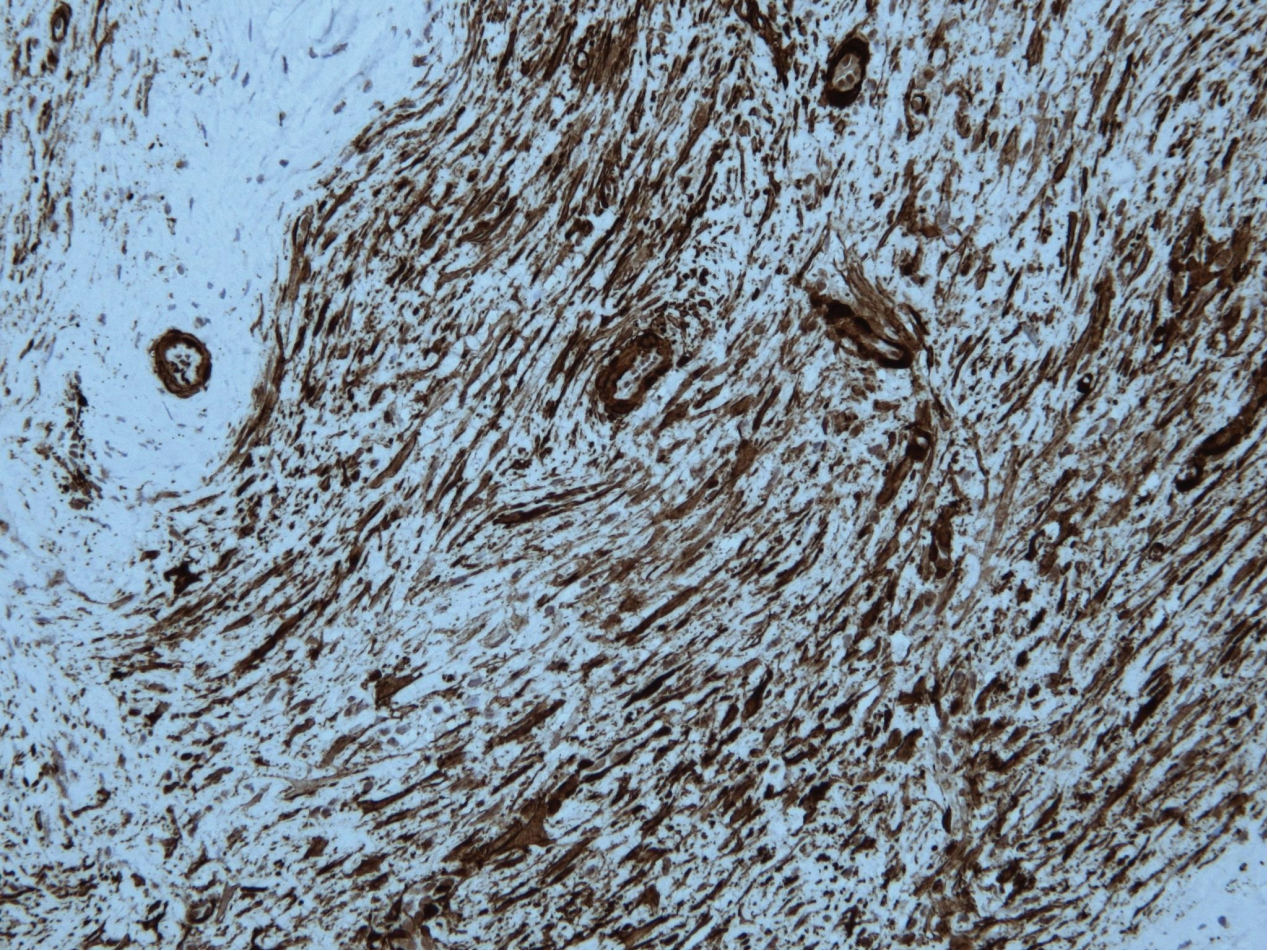
SMA staining.

**Figure 3:**
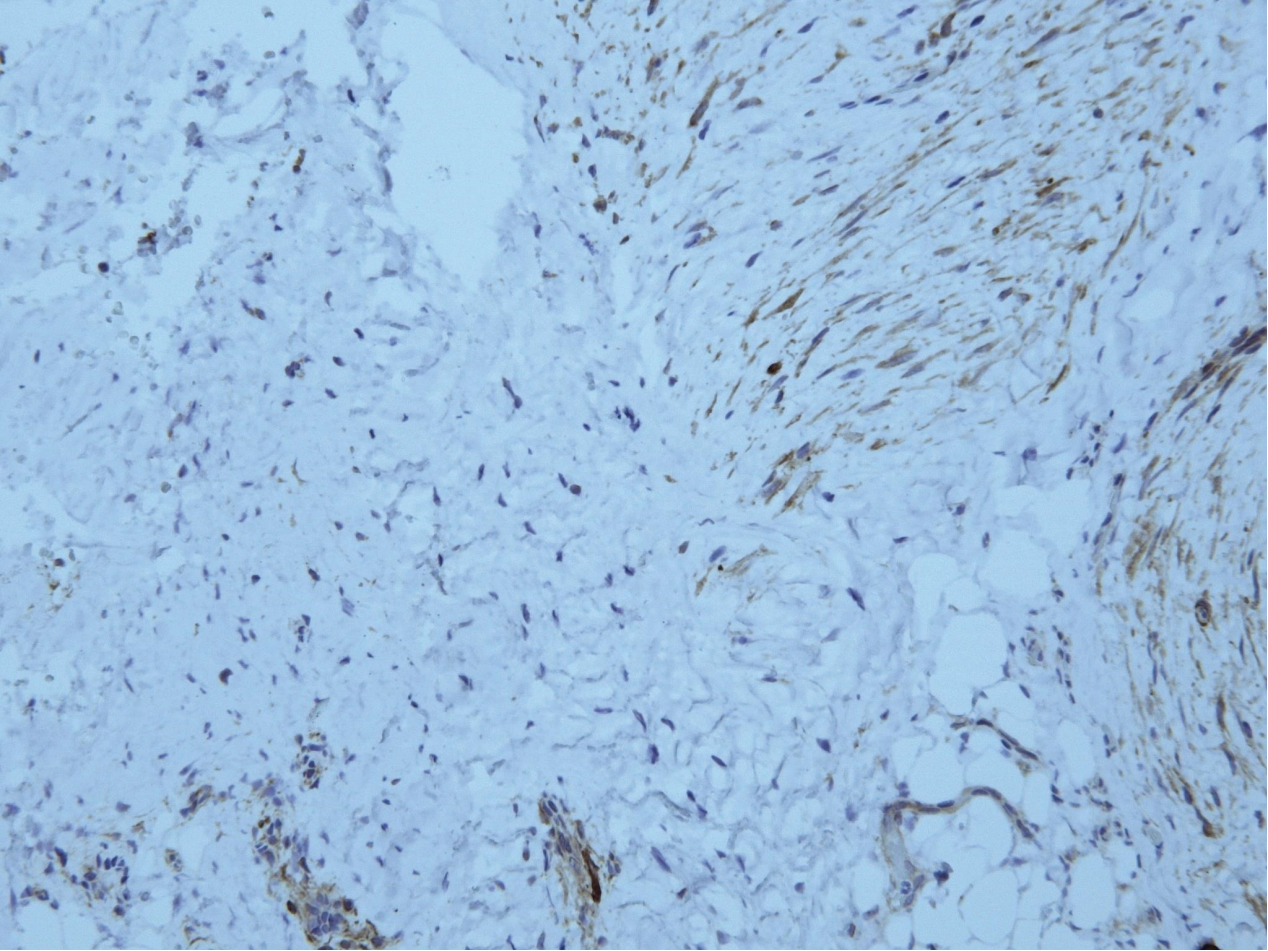
H‐caldesmon.

**Figure 4:**
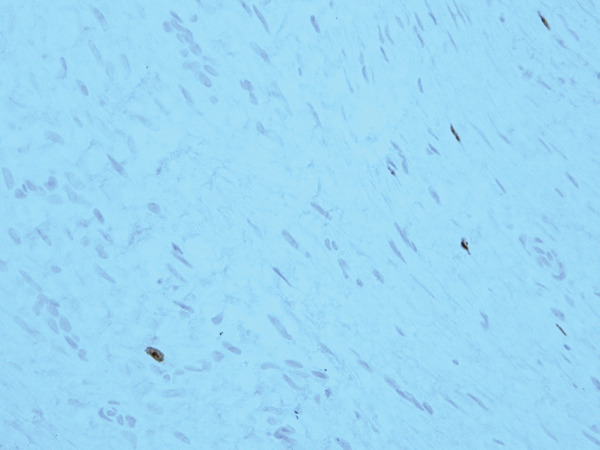
Ki‐67.

Subsequent histological examination confirmed Dupuytren′s disease, characterized by dense collagenized connective tissue formed by spindle cell lesions arranged in irregular fascicles with low cellularity. The cells were slightly variable in size, without any atypical features or mitotic activity (Figure 1). Immunophenotype of the tumor cells was as follows: SMA, H‐caldesmon and Ki‐67 were positive; beta‐catenin, desmin, S100, CD34, EMA, and CK‐AE1/3 were negative (Figures 2, 3, and 4).

**Figure 5:**
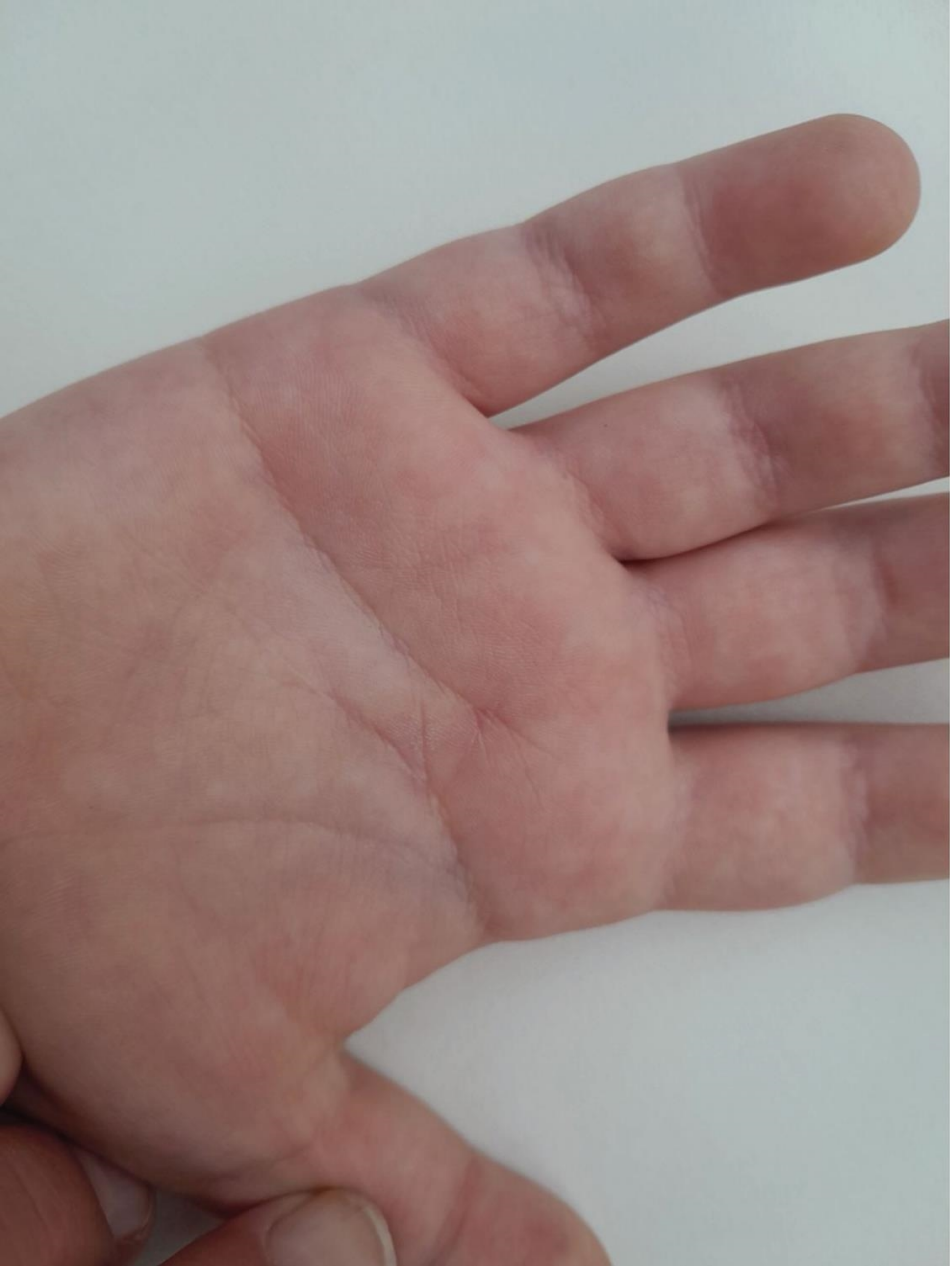
Photo of the treated hand at 2‐year follow‐up—palmar view.

**Figure 6:**
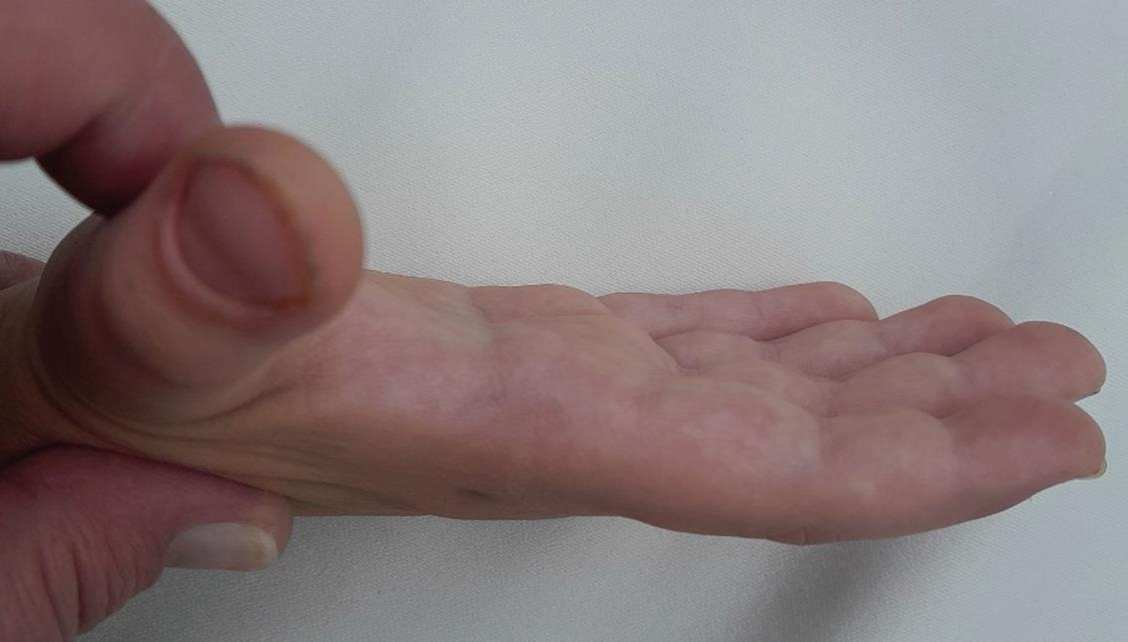
Photo of the treated hand at 2‐year follow‐up—lateral view.

At the time of writing, 2 years after surgery, the functional outcome of our patient remains excellent. No recurrence of the disease has been observed and full range of motion is preserved in the affected hand (Figures 5 and 6). Given the nature of the disease, however, the authors acknowledge that even a 2‐year follow‐up is not sufficient to allow definitive discharge from follow‐up.

### 2.1. Discussion and Literature Review

Although common in adults, Dupuytren′s disease is very rare in children. In the literature search using the PubMed database with MeSH terms “pediatric,” “paediatric,” “Dupuytren,” “Dupuytren′s,” “palmar,” and “fibromatosis” together with screening of reference lists of the identified publications, we found a total of 32 reports of pediatric cases of Dupuytren′s disease published between 1950 and 2025, mostly in the form of single case reports (Table 1). Given the heterogeneity and variable level of detail in previously published pediatric cases, our analysis is based on data available from accessible reports and secondary literature reviews, and should be interpreted accordingly.

**Table 1:** Summary of previously published cases of pediatric Dupuytren′s disease with reported demographic data, clinical presentation, treatment modalities, follow‐up duration, and outcomes.

Author	Age	Gender	Duration	Treatment	Recurrence	Outcome
Goetzee and Williams [1]	14 years	M	3 years	Fasciectomy	U	Full ROM
Hueston [2]	12 years	M	U	Fasciectomy	U	U
Rosenfeld et al. [3]	13 years	F	3 years	Excision	U	U
Berger and Gurr [4]	2 years	M	13 months	Segmental fasciectomy	U	U
Urban et al. [5]	9 years	M	6 months	Fasciectomy	Yes	Recurrence requiring two subsequent surgeries
						New disease discovered in left hand requiring dermofasciectomy
Urban et al. [5]	10 years	M	U	Excision biopsy	U	U
Foucher et al. [6]	10 months	M	Congenital	Cord excision	None at 27 months	Full ROM
						No recurrence or progression of disease
Rhomberg et al. [7]	2.5 years	F	6 months	Cord excision	Yes	Required two subsequent surgeries due to flexion contractures
						Good end result with 10° extension lag
Rhomberg et al. [7]	10 years	M	Congenital	Fasciectomy	None at 2 years	Full ROM
						No progression
Rhomberg et al. [7]	9 years	M	1 year	Partial fasciectomy and arthrolysis	Yes	Forearm amputation due to sarcoma
						Myoelectric prosthesis
Mandalia and Lowdon [8]	10 years	M	1 year	Excision biopsy	U	U
Bebbington and Savage[9]	6 months	M	U	Excision biopsy	None at 21 months	No functional disturbances of the hand
						Full active flexion. 10° loss of active extension of D4
Fetsch et al. [10]	12 years	M	U	Excision biopsy	U	U
Fetsch et al. [10]	U	U	U	U	U	U
Lee et al. [11]	12 years	M	U	Fasciectomy	U	U
Fernandez‐Garcia et al. [12]	12 years	F	U	Dermofasciectomy	None at 13 months	Resolution of all symptoms
						No persistent disturbances
Marsh and Kelly [13]	8 years	M	1 year	Fasciectomy	None at 12 months	Full ROM
						No recurrence or progression of disease
Tiong et al. [14]	12 years	M	18 months	Fasciectomy	Recurrence after 1‐year post‐op	A subsequent dermofasciectomy due to contracture
Usmar and Peat [15]	8 years	M	6 months	Excision biopsy	None at 3 months	U
Korambayil and Padikala [16]	4 months	M	U	Excision biopsy + fasciectomy	U	U
Kraus et al. [17]	7 years	F	1 year	U	None at 12 months	Resolution of all symptoms
						No persistent disturbances
Gary and Santamarina [18]	6 years	M	3 months	Fasciectomy	None at 6 months	Full ROM
						No recurrence
Zheng and Liu [19]	31 months	M	Congenital	Fasciectomy	None at 21 months	No functional disturbance
						Full ROM except for limited abduction of ring finger
Spyropoulou et al. [20]	10 years	M	4 months	Dermofasciectomy	Yes	Recurrence at 4 months requiring a subsequent surgery
						No further recurrence at 1 year after second surgery
Żyluk and Walaszek [21]	17 years	M	11 years	Fasciectomy	None at 1 year	Slight contracture of fifth finger associated with scar formation
						No recurrence
Garcia‐Mata and Duart‐Clemente [22]	13 years	F	2 years	Dermofasciectomy	None at 16 years	Full active and passive ROM
Civan et al. [23]	9 years	M	U	U	Yes	U
Kleinsorgen et al. [24]	6 months	F	4 months	Fasciectomy and arthrolysis	Yes	Recurrence of contracture with a requirement of subsequent surgery at 19‐months post‐op No further recurrence at 19 years
Beecher et al. [25]	8 years	M	6 months	Dermofasciectomy	U	U
Kapay et al. [26]	10 years	M	Few months	Excision biopsy + fasciectomy	U	U
Lee et al. [27]	14 years	M	1 yearr	Triamcinolone injections	None at 2‐year postsurgery	No improvement on intralesional injections
						Subsequent fasciectomy with excellent result—full ROM
Soltani et al. [28]	14 years	M	1 year	Segmental fasciectomy	None at 7 months	Full ROM

Abbreviations: M/F, male/female; ROM, range of motion; U, unavailable data.

It should be noted that Ushijima et al. [29] in his 1984 review reported 42 cases of palmar fibromatosis with ages ranging from 15 to 76 years at the time of surgery; however, these have not been included in the present literature review, as the exact number of pediatric cases is not stated in the article. In addition to two original cases published by Urban in his 1996 article, five further cases of histologically proven Dupuytren′s disease of hand in the pediatric population were reported by Urban et al. [5] in the same article, based on personal communication with Fletcher. These specimens were analyzed in a specialist oncological unit and were reported without any clinical data. Fourteen of the cases in our review are included in a systematic review published in 2015 by Izadpanah et al. [30]. Twenty‐one cases were reported in the literature review by Beecher et al. [25] in 2021. An additional nine cases were published between 2005 and 2024 by Fetsch et al. [10], Lee et al. [11], Żyluk and Walaszek [21], Civan et al. [23], Kleinsorgen et al. [24], Kapay et al. [26], Lee et al. [27], and Soltani et al. [28]. We now provide a description of another histologically confirmed case of palmar fibromatosis present in a child that was treated surgically.

Despite the small numbers of cases, Izadpanah′s systematic review noted that within follow‐up periods, ranging between 3 and 27 months, 50.0% of patients (*n* = 7) experienced positive outcomes without any functional impairment in the affected hand. Treatment with excision biopsy (two patients), dermofasciectomy (one patient), cord excision (one patient), fasciectomy (two patients), and an unspecified surgical intervention (one patient) was chosen for these patients. Poor functional outcome was noted in three patients (21.0%), all of which had recurrence of disease. These patients underwent fibrotic band excision (one patient), partial fasciectomy with arthrolysis (one patient), and fasciectomy (one patient). In four patients, the functional outcome was not stated. Excellent outcome was noted in five of the other published cases, which had follow‐up periods from 12 months to 13 years [30].

Of the 32 cases of reported pediatric Dupuytren′s disease, the mean age of presentation was 8.9 years (range congenital to 17 years), with a mean symptom duration of 1.8 years (range: 3 months–11 years).

There was a clear male predominance, with 25 of 31 cases (80.6%) being male.

Of the 29 cases with a known first‐line surgical treatment, 16 patients (55.2%) were treated with fasciectomy, including 2 patients (6.9%) who underwent fasciectomy combined with arthrolysis and 2 patients (6.9%) who underwent fasciectomy combined with cord or nodule excision. Eight patients (27.6%) were treated with excision alone, whereas four patients (13.8%) underwent dermofasciectomy. In one case (3.4%), the patient was treated with triamcinolone injections.

The mean reported follow‐up was 27 months (range: 3 months–18.2 years) with an adjusted mean of 14.3 months, if a single outlier follow‐up of 16 years is excluded.

Where data are available to the authors, disease recurrence was reported in 7 of 20 patients (35%), whereas 13 patients (65%) remained free of reported recurrence. In one patient (5%), the disease manifested also in the contralateral hand.

In comparison with our systematic review, our patient′s data are consistent with several established characteristics of pediatric Dupuytren′s disease, including male sex, age at presentation, and nodular mass as the presenting symptom. Additionally, our patient′s only apparent potential cause of disease was trauma. We treated the patient with nodule excision and limited fasciectomy (vs. 27.6% of nodule or cord excision alone, or 6.9% of combined nodule or cord excision with fasciotomy). To date, our patient remains free of recurrence, consistent with the 65% of patients in the available literature.

Whereas Dupuytren′s disease most commonly presents as a palmar nodule or cord arising from the palmar fascia, often causing progressive, painless finger flexion contracture without systemic symptoms, other conditions may share certain clinical features.

Burn contractures are associated with a clear history of thermal injury and result in discolored scar tissue and contractures rather than a fascial nodule or cord. Infantile digital fibromatosis usually affects fingers and toes, presenting as firm dermal nodules on the lateral or dorsal aspects of the digits rather than in the palm. Camptodactyly presents as a congenital or developmental flexion deformity, often familial, bilateral and affecting the proximal interphalangeal joint without involvement of the palmar fascia. Tenosynovial giant cell tumor usually presents as a painless, slowly enlarging nodule arising from a tendon sheath and may be distinguished on MRI by the presence of hemosiderin deposition. Fibroma of the tendon sheath is a well‐circumscribed slow‐growing mass attached to a tendon sheath and usually lacks both contracture and fascial cord formation. Infantile fibrosarcoma is a rapidly enlarging mass which may be painful, cause skin changes, ulceration or neurovascular compromise. Scleroderma is a skin thickening disease often extending beyond the palm, associated with systemic manifestations such as Raynaud phenomenon and digital ulcers, and is often bilateral.

## 3. Conclusion

Although trauma including burns, infantile digital fibromatosis, camptodactyly, tenosynovial giant cell tumor, fibroma of the tendon sheath, infantile fibrosarcoma, and various connective tissue disorders including scleroderma and various congenital disorders remain among the more common diagnoses, which should be excluded at first, our finding further reinforces the case for including Dupuytren′s disease in a differential diagnosis for palmar nodule or finger contracture in the pediatric population. It is also consistent with the current suspected risk factors for Dupuytren′s disease in children, such as male sex, previous hand trauma, and Caucasian race. Surgical treatment yields good results and is advisable for histological verification, especially in ruling out the malignant potential of the mass.

## Funding

This study was supported by the Ministerstvo Zdravotnictví Ceské Republiky (10.13039/501100003243) (00064203).

## Disclosure

The authors confirm that they are authors of the proposed manuscript. Full responsibility for the accuracy and integrity of the manuscript rests with the authors.

## Ethics Statement

The study was performed in accordance with the Helsinki declaration. Publication of the paper was approved by the Ethical Committee of the Second Faculty of Medicine of Charles University and Motol and Homolka University Hospital.

## Consent

The authors declare that they have obtained the parental consent for publication in both English and the native tongue of the parents.

## Conflicts of Interest

The authors declare no conflicts of interest.

## Data Availability

The data that support the findings of this study are available from the corresponding author upon reasonable request.
